# Supplementary material for: Understanding the burden of poor mental health and wellbeing among persons affected by leprosy or Buruli ulcer in Nigeria: A community based cross-sectional study
Source: PLoS One. 2024 Jun 17;19(6):e0304786. doi: 10.1371/journal.pone.0304786 (PMC11182497; doi:10.1371/journal.pone.0304786)
Supplement: Attachment — Submitted filename: Response to Reviewers.pdf [file pone.0304786.s004.pdf]

Reviewer’s comments and response from Authors

Introduction

Authors should please list the studies that dealt with mental health and collectively elaborate on the findings of the studies to limit the bulk of the introduction and improve the clarity of their manuscript. The introduction needs to be fine-tuned properly for better comprehension of the available information. Authors still need to work on information flow.

Author response

Thanks for the comment. Very few studies focused on mental health of persons affected by leprosy or BU in Nigeria. Those found were included. Studies from other parts of the world especially India were cited in great numbers in the discussion. Authors however worked on the information flow in the Introduction section. I hope the manuscript is acceptable in this present form.

Reviewer’s comment

Methods

It is a fact that Nigeria is the most populous nation in Africa but what we need under the study setting is for you to state and cite the country’s population for readers to have an idea of the population.

Author response

This has been included. See study setting.

Reviewer’s comment

Study population

Clearly state the inclusion and exclusion criteria for instance “the inclusion criteria for the study were persons affected with leprosy or BU who were registered…………………………………………while the exclusion criteria for our study were……………………………………”.

Line 147: you made mention that age was not a barrier to inclusion in your study, why did you put age as part of the inclusion criteria in your initial statement? Please, delete age in your inclusion criteria if it was not used in the selection of participants. Be precise in your statements.

Author response

First the individuals affected by leprosy or Buruli ulcer must have been registered for treatment under the care of NTBLCP which is the agency of Government responsible for the management of tuberculosis, leprosy and Buruli ulcer in the country. After this, age was applied differently for the inclusion of persons affected by leprosy or Buruli ulcer in the study.

'For persons affected by leprosy only those who were 18 years and above were included in the study. However, because of the epidemiology of BU in the country, age was not a barrier for inclusion of persons affected by BU in the study.'

Reviewer's comment

Line 152 Sample size determination and sampling technique

Please delete determination of sample size as indicated by earlier reviewer

Under sample technique, you can state the sampling method and the method of recruitment of participants in the study. It is also good to state the total number of participants that participated in the study.

Author response

This has been included. See sampling technique.

Reviewer's comment

The authors made mention of limited bias in the study due to the fact that areas with high risk of leprosy and buruli ulcer were selected for the study to improve the quality of data that will be obtained. Also, they made mention of the number of study participants. This should be clearly stated under the method and not just in the response to the reviewers bearing in mind that there could be possibility of bias even with large samples since the participants were not randomly sampled.

Author response

Thanks for the observation.

The limitations of the study have been improved upon.

Reviewer's comment

Line 159 Data collection tools/methods please clearly state the website of the data collection tools used in the study if available

Author response

Included. Thanks

Reviewer's comment

Line 200 please write the heading in full. You can abbreviate in bracket

Author response

This has been done. My appreciations.

Reviewer's comment

Line 239-241; the duration of the study should not be stated under ethical clearance. It should be stated under the study design.

Author response

Included.

Reviewer's comment

Results

Please ensure that the total number of participants/respondents is indicated in all the titles of the tables presented. Example; Assessment of anxiety symptoms among persons (n=635) affected by leprosy or Buruli ulcer in Nigeria using GAD7. You can delete the n=635 within the tables.

Author response

Done

Reviewer's comment

Line 301; χ^2=0,010 please replace the comma with a dot in stating the Chi square value

Author response

Corrected. Thanks.

Reviewer's comment

Line 304, Table 7, please appropriately place the 1 value for the GAD 7 score compared with the GAD 7 score

Author response

Done

Reviewer's comment

Discussion

Line 342 From the results of this study also? Why not write "the study also reveals…..".

Author response

Corrected.

Reviewer's comment

Please endeavor to edit the entire manuscript properly.

Author response

I have done that. Thanks

Reviewer’s comment

The discussion should follow the pattern to which your results were presented. Your discussion need to be concise and articulated to ensure the flow of the discussion. Eg studies in India have indicated the range of depressive disorders from 30-53% (--citations). I suggest that repetition of points should be avoided, for example Line 359-360, 433-434, 446-447; all referring NTDs as a risk factor in developing poor mental health. Regarding the possible effect of COVID-19 accruing to the high level of depression, anxiety and poor mental health in this study can be presented in a concise manner.

Author response

Corrected.

Reviewer’s comment

Line 447-450 should be the limitation of the study. It should be the last paragraph of the discussion. Purposive sampling is a limitation based on sampling method. Preferably, probability sampling should be used for the generalization of results and to eliminate bias. Could it be that those areas that were not sampled, if sampled, the results obtained could have differed based on sociocultural and economic differences? The sample size of the study was large which might have aided in the research but may not completely rule out elements of bias. Other similar studies that were cited, did they use probability sampling? The issue of selection bias may not be totally ruled out due to the nature of the sampling involved. I suggest you mention it as part of the limitation of your study.

Author response

The limitations of the study have been reviewed.

Thanks

Reviewer’s comment

Conclusion

Line 453 Please rephrase, do not start a sentence with “with”.

Author response

Done.
