# Supplementary material for: Understanding the burden of poor mental health and wellbeing among persons affected by leprosy or Buruli ulcer in Nigeria: A community based cross-sectional study
Source: PLoS One. 2024 Jun 17;19(6):e0304786. doi: 10.1371/journal.pone.0304786 (PMC11182497; doi:10.1371/journal.pone.0304786)
Supplement: Attachment — Submitted filename: Response to Reviewers.pdf [file pone.0304786.s005.pdf]

Reviewer comment and response from Authors

Reviewer # Comment
Dear authors, the topic of your study has been registered as a study protocol in which authors included except some of them.
STUDY PROTOCOL
A Cluster Randomized Trial for Improving Mental Health and Well-Being of Persons Affected by Leprosy or Buruli Ulcer in Nigeria
A Study Protocol
Ekeke, Ngozi1; Ossai, Edmund Ndudi2,; Kreibich, Saskia3; Onyima, Amaka4; Chukwu, Joseph1; Nwafor, Charles1; Meka, Anthony1; Murphy-Okpala, Ngozi1; Henry, Precious1; Eze, Chinwe1
link:
https://journals.lww.com/ijmy/fulltext/2022/11020/a_cluster_randomized_trial_for_improving_mental.1.aspx
Therefore, objectives are mentioned in the study protocol, I expect they will be addressed and published (I do not know when). What your study adds different from the planed study? It seems that authors need to publish the baseline information of patients. I think this is not recommended as it may be considered fragmenting one paper to multiple separate paper for the matter of publication. It is better to conduct and publish the trial timely. In my view the present manuscript should be rejected due to the points raised above.

Author response

Dear Editor,

Thank you for the comment. The Reviewer is right and indeed the Authors of the manuscript did not hide this fact during the submission process. This was when we were asked if the manuscript has been submitted to any of the PLOS Journals. We admitted that when we submitted the baseline result to PLOS NTD for publication, the Editor and Reviewers advised and guided the Authors to publish the result as a cross-sectional study which we eventually adhered to.

We have also observed that with the comments of the Reviewers from PLOS NTD, the presentation of the results of this study as submitted is very different from what was included in the study protocol. The outcome variable of this study as presented is ‘poor mental health/wellbeing’ which is entirely a new concept. Another observation is that there are very few studies in Nigeria that focused on the mental health and wellbeing of persons affected by leprosy or BU and none again has as much sample size and spread (five states and two geo-political zones in Nigeria).To a large extent, this study as presented brought to the fore the huge burden of poor mental health/wellbeing among persons affected by leprosy or BU in Nigeria. The focus of the result as contained in the study protocol will be on the effect of the intervention.

All these points as noted above is not to justify any wrong doing on the part of the Authors but to plead with the Editor and Reviewers of PLOS ONE Journal to see the contribution of this study

to the body of knowledge especially as it pertains to Nigeria and approve that the peer review of this manuscript should continue.
