# Supplementary material for: Understanding the burden of poor mental health and wellbeing among persons affected by leprosy or Buruli ulcer in Nigeria: A community based cross-sectional study
Source: PLoS One. 2024 Jun 17;19(6):e0304786. doi: 10.1371/journal.pone.0304786 (PMC11182497; doi:10.1371/journal.pone.0304786)
Supplement: Attachment — Submitted filename: Response to Reviewers.docx [file pone.0304786.s002.docx]

**Reviewer comments and Response by Authors**

**Reviewer comment**

The topic of your research is of importance. However, both reviewers express some concerns, e.g. concerning the lack of objectives of the study. Moreover, reviewer 1 points out that presenting the baseline data without describing the cluster RCT does not make sense. I agree with this appraisal. As the authors do not present any information on objectives, intervention, sampling, clusters and randomisation, this article is of very limited value.

As a personal response, I would like to add that there is no data on the validity of the applied questionnaires in the domestic language. Further, the high prevalence of clinical anxiety and depression is due to the cut-off score chosen - most studies use a score of 10 for clinical levels of anxiety and depression. So before relating the high prevalence rate to the aftermath of the COVID-19 pandemic, the authors might want to focus more strongly on methodological aspects.

Author response: Two of the three validated questionnaires used in the study have been extensively used in Nigeria except WEMWBS. The authors did a validation of this tool (WEMWBS) in three of the major local languages in the study area before being applied in the study. The results of these validation studies will soon be published.

In truth, the authors may have overlooked this good observation by the Editor that the other two questionnaires, PHQ9 and GAD7 have not been validated in the local languages in Nigeria. The authors apologize for this oversight.

The results of PHQ9 had two different categorical approaches. First the score was classified into five categories. This enabled us to compare very well with the result of the study in Bangladesh. (See lines 327-335). The second approach was classification based on presence and absence of depressive symptoms. We agree with the Editor that a score of 10 denotes moderate depression while scores of 5-9 stand for mild depression. Furthermore, correlation analysis was used to compare the findings of total scores from PHQ9, GAD 7, WEMWBS, Oslo social support scale and age of the respondents since they are all continuous variables..

**Reviewer comment**

Reviewer #1: The work of Ossai et al. „Cluster randomized trial for improving mental health and wellbeing of Persons affected by Leprosy or Buruli ulcer in Nigeria: Baseline results“ describes a survey in the context of a cluster-randomised study in Nigeria on people suffering from leprosy and Buruli ulcer. The data is from a baseline survey. Questionnaires on depression (PHQ-9), anxiety (GAD-7) and quality of life (WEMWBS) are used for a total of N = 635 persons suffering from the above-mentioned diseases (intervention group N = 319, control group N = 316). It was found that almost 90% of the respondents had depressive symptoms, almost 80% had anxiety symptoms and more than half of the respondents had a low quality of life. There was a strong correlation between depressive and anxious symptoms, a weak negative correlation between depressive and anxious symptoms and quality of life. The presence of social support had a positive effect on depressiveness.

According to the reviewer's assessment, the manuscript must be rejected for the following reasons:

1) This study has already been registered as a trial. The description of the study and the study design is therefore no longer of interest. A differential description of an intervention group and control group in the form of baseline results without results of the planned intervention is of little to no interest to the readership. What should she derive from this? Thus, in a narrower sense, this is a cross-sectional study.

Author response: This advice is the basis for presenting the study as a cross-sectional study while we await the result of the intervention.

**Reviewer comment**

2) For this, however, the introduction is not purposefully structured. It deals with health-related needs, the effects of the COVID pandemic and, very briefly, the psychological burden of people suffering from leprosy and Buruli ulcer. The aim of the study is stated as being to provide access to quality and affordable health care. With this in mind, the authors state that the study was designed as a cluster-randomised trial. No research question and no objective are stated for the presentation of the baseline results.

Author response: This has been taken care of with the presentation of he results as a cross-sectional study.

**Reviewer comment**

3) The question arises in which way the results obtained in the cross-section go beyond the previous literature on mental comorbidity of leprosy and Buruli ulcer and which extended findings go beyond other studies that are completely inadequately presented in the introduction.

Author response: The results are presented differently based on the advice of presenting the work as a cross-sectional study.

**Reviewer comment**

4) For the evaluation, questionnaires commonly used in the field of mental health will be used. Their reliability and validity are not supported with literature according to the standards.

Author response. The PHQ9 and GAD7 questionnaires have been used extensively in Nigeria. WEMWBS does not have that history hence the authors validated the questionnaire first in three local languages in the study area. The results of the validation will soon be published.

**Reviewer comment**

5) Comparisons between the control group and the intervention group are of little or no interest; if so, comparisons with the normal population (possibly from the manual) or with other studies or extended findings using regression analyses should be sought.

Author response: This is presently presented as a cross-sectional study.

**Reviewer comment**

6) The discussion would have to establish a connection between the descriptive results and previous studies. It remains to be assumed that the results do not sufficiently go beyond the current state of knowledge on psychological distress in leprosy and Buruli ulcer (unless the authors can demonstrate this), which is why a format of a short communication should be considered in a revision.

Author response: This study could serve as one of the largest studies that focused on mental health of persons affected by leprosy or Buruli ulcer in Nigeria. This is one of its relevance. Again, it is the baseline of a cluster randomized study which is also the first of its kind in Nigeria of which seasoned editors advised that the result be presented as a cross-sectional study. In as much as the results may not go beyond the current state of knowledge on psychological distress in leprosy and Buruli ulcer, the authors are of the opinion that the study should be published as a cross-sectional study at least to add and support the findings from Nigeria. Moreover, the results of the intervention is being awaited. Publishing this study as a short communication will not be of good to the objectives of the study and it will be a poor foundation to the cluster randomized approach.

**Reviewer comment**

7) Further note: The Corona Pandemic is not "a trauma" as named several times in the manuscript. This is not correct in the psychotraumatological sense. The reviewer would recommend refraining from using this formulation, as it does not apply in the psychotraumatological sense. Should the editors consider revising and adapting the manuscript test, I am prepared as a reviewer to provide a more detailed analysis.

Author response: The COVID pandemic was noted as a possible limitation of the study.

**Reviewer comment**

Reviewer #2: The objectives are not explicitly stated in the paper. The aim of the study is the prevalence of leprosy and Buruli's ulcer in Nigeria. The study design is appropriate  and he sample size is clearly described. The sample size is adequate for interventions like these and must have been elaborated in the protocol paper. The results mention statistical comparisons between the study and control groups using p-values and correlations between variables. Cannot commnent on the correctness of the analysis used. For ethics approval , I am presuming that it would be in the protocol paper. Good to mention it here too .

Author response: Thanks. The ethical approval is included in this submission.

**Reviewer comment**

Reviewer #2: The results are presented clearly. The proportion of respondents with depressive disorders was much higher compared to the control group , but there is no explanation for this. Also the potential impact of COVID is mentioned but also says this caanot be deduced from the results , Then other possible factors for this difference must be explored. The difference in education can confound the results.

Author response: The results are now presented in a different form since this presentation is now based on a cross-sectional study design.

**Reviewer comment**

Reviewer #2: COVID pandemic would have had a significant impact on mental health and should have been accounted for in the baseline mental health status. The high prevalence of depression and anxiety should be interpreted with caution. This study is a part of a larger intervention. Results from that study will inform mental health practise - it is presumed.

Author response: This is noted with thanks.
