# Supplementary material for: Understanding the burden of poor mental health and wellbeing among persons affected by leprosy or Buruli ulcer in Nigeria: A community based cross-sectional study
Source: PLoS One. 2024 Jun 17;19(6):e0304786. doi: 10.1371/journal.pone.0304786 (PMC11182497; doi:10.1371/journal.pone.0304786)
Supplement: Attachment — Submitted filename: Response to Reviewers comments.docx [file pone.0304786.s003.docx]

- [Drafts1.1K](https://mail.yahoo.com/d/folders/3)

**Reviewer’s comments and responses by Authors**

Academic Editor,

TGGHJJKCVXVXBZXNZNZNZNZNZTTTQQQRRRRR

**Reviewer’s comment**

Title

Where exactly in southern Nigeria were these studies conducted?

This should be reflected in the title as well as the methodology. For the methodology, state the specific locations were participants were recruited, which communities? And under which Local Government Areas and States? This needs to be extrapolated for clarity. State the names of the communities, LGAs and States involved in the study.

**Author response**

**Many thanks for this comment. The states and local government areas have been included. The communities may be very difficult to define. This is because the NTBLCP which is the agency of Government in charge of leprosy and Burruli ulcer activities are structured along the three tiers of government in Nigeria which are the Federal, State and Local Government Areas.**

**Reviewer’s comment**

Introduction

I suggest that you start the introduction from the global effect of mental health regarding leprosy and Buruli ulcer, followed by the continental aspect and in Nigeria and finally the study area.

**Author response**

**This has been included. See lines 106-108.**

Methodology

**Reviewer’s comment**

Study population; line 130-133- clearly state the inclusion criteria and exclusion criteria.

Were there any chances of introduction of selection bias in your studies? You made mention that you used purposive sampling in selection of study sites. Did your findings fully reflect the entire Nigerian situation? How were you able to reduce bias in your studies? How were you able to deal with issue of confounders to ensure that your result reflects what you intend to achieve. Please, categorically state it in your methodology.

**Author response**

**The inclusion and exclusion criteria have been included. See study population. The officials of NTBLCP were fully involved in the study and they have a register of all persons diagnosed with leprosy or BU in all the LGAs of the country. So selection bias was not a problem in the study. The LGAs were purposively selected based on case notifications between 2014 and 2018. This was purely to ensure maximal impact and this may not have affected the results of the study**

**Reviewer’s comment**

RESULTS

Please label all the tables properly, eg Table 1: Socio-demographic characteristics of the respondents (n=635)  ------(location) in Nigeria

**Author response**

**This is noted. Corrected.**

**Reviewer’s comment**

Discussion

Limitations of the study should be the last paragraph of your discussion. Please delete the heading “limitations”,

**Author response**

**The heading ‘limitations’ has been deleted.**

**Reviewer’s comment**

Conclusion

Be precise by stating in percentage the burden of poor mental health/wellbeing among persons affected by leprosy or Buruli ulcer in your study.

**Author response**

**Included**

**Reviewer’s Comment**

Reviewer #1: 1. I would suggest using term 'Mental Health Conditions' or 'Mental Distress' instead of 'Poor Mental Health' throughout the manuscript including the title. The term 'poor mental health' would increase stigma.

**Author response**

**This suggestion is appreciated. However, the outcome variable in this study is poor mental health/wellbeing. This is because the variable was derived from the use of three validated questionnaires including PHQ9, GAD7 and WEMWBS. That is why it appears that poor mental health/wellbeing may be the most appropriate to represent this variable.**

**Reviewer’s Comment**

2. In Introduction section- It would be good to give detailed definition of the Leprosy and BU. This is important for the audience who does not have medical background. Also, it is important to mention the prevalence of Leprosy & BU in the study settings/geographical area.

**Author response**

This has been included, see lines 74-76.

**Reviewer’s Comment**

3. It is not clear that why researchers chose to study prevalence of MH conditions among persons with Leprosy or BU only and not from all NTDs? The aim and objectives of this study are not clear and need to be clearly mentioned. Are researchers already involved in MH care for persons with Leprosy and BU? The way authors have mentioned need of MH intervention among persons with Leprosy is not very detailed one, and very superficial. Also, authors have mentioned effect of COVID 19 pandemic on persons with Leprosy but what about persons with BU.

**Author response**

This study was focused on persons affected by leprosy or Buruli ulcer. In-fact, the study prompted another one which included other NTDs. Objective: This study was designed to assess the mental health and wellbeing of persons affected by leprosy or BU in Nigeria. In Nigeria, mental health services are not included in the health services for persons affected by leprosy or BU and the finding of this study should suggest the need for such services. The literature has much on the effect of COVID-19 pandemic on persons affected by leprosy unlike BU.

**Reviewer’s Comment**

4. There needs to be clarity around validity of each scale in local language and culture. Also, it would be better to mention Cronbach's Alpha (Coefficient) for mentioned scales. Please mention validity for Oslo Social Support Scale.

**Author response**

**We did not include validity for Oslo Social Support Scale in our study but the tool has been used several times in our study setting.**

**Reviewer’s Comment**
5. Similarly, inclusion and exclusion criteria for this study need to be mentioned.

**Author response**

**The inclusion and exclusion criteria of the study have been included. See study population.**

**Reviewer’s Comment**
6. Please mention training details for data collectors.

**Author response**

**Training of research assistants centered on the use of the study tools, obtaining informed consent and the need for confidentiality and also privacy when collecting information from the respondents, etc. Officials of NTBLCP who accompanied the research assistants identified persons affected by leprosy or BU for interview.**

**Reviewer’s Comment**

7. For consent process- please mention details of consent procedure for persons who cannot read or write. Whether authors followed ethical guidelines for this group?

**Author response**

The process of obtaining informed consent took place using the local language so an interpreter was present and also a witness. When the process was over, the respondents will explain all that they understood from the process. It is only when this was judged as adequate that the responded thumb printed on the informed consent form followed by the signature of the witness.

**Reviewer’s Comment**

8. The manuscript will need through proofread for the language. Line no 76- need to say - infections those lead to a disability instead the mentioned term. Line 98- it needs to be 'quarantine' and not 'quarantining'.

**Author response**

This has been taken care of. Many thanks.

**Reviewer’s Comment**

9. Also, it would be good to mention if Authors will use this data to push any policy change at local or state level or wanted to further use this data to design community-based MH intervention for persons with Leprosy and BU.

**Author response**

The reviewer is right. A community based MH intervention for persons affected by leprosy or BU is on-going.

**Reviewer’s Comment**

10. Is this the first study from Nigeria / Africa measuring prevalence of MH conditions among person with Leprosy and BU? If yes, then this need to be mentioned clearly.

**Author response**

**This is not the first study from Nigeria that measured the prevalence of MH conditions among persons affected by leprosy or BU. However, it could be the first that involved such a high number, (n=635) and spread. The study took place across six states in two geo-political zones of the country.**

**Reviewer’s Comment**
Reviewer #2: 1. Please ensure that, before resubmission, the manuscript is carefully checked for English language and grammatical errors. It is important that the message being conveyed in the manuscript is as unambiguous as possible.

**Author response**

**Many Thanks for the comment. Grammatical errors noticed have been corrected.**

**Reviewer’s Comment**

3. Introduction can be shortened to make it more clear

**Author response**

**Thanks. Information is noted.**

**Reviewer’s Comment**

4. The discussion is to long and it lakes coherence, try to make it short and precise

**Author response**

**Noted. Done,**

**Reviewer’s Comment**

5. in the study area try to show us your study area with regard to the number of hospitals with its capacity to give service particulary poor mental health

**Author response**

**The observation that hospitals with such capacity are few and concentrated on urban areas which are very far from these communities prompted the MH intervention that is ongoing which is community based by making use of available human resources who are trained for that purpose.**

**Reviewer’s Comment**

6. in your background section try to illustrate the over all burden of poor mental health among the specified problems and clearly state the gaps and how you are going to fill it.

**Author response**

See lines 90-97 and lines 93-112.

**Reviewer’s Comment**
7. please ommit the sentence, Nigeria is the popular countary,

**Author response**

We indicated that, ‘Nigeria is the most populous country in Africa,’ and that is a fact.

**Reviewer’s Comment**
8. try to delet the word skin from skin tropical neglected..... start with tropical neglecte.

**Author response**

**Corrected.**

**Reviewer’s Comment**
9. you selecte the sample purposively, so the result could not generalize the total population. pls delete sample size determination heading.

**Author response**

NTBLCP is the agency of Government in Nigeria responsible for TB, leprosy and BU activities in Nigeria. We used their data to select ten LGAs in southern Nigeria that reported the highest cases of leprosy and BU between 2014 and 2018. These ten LGAs were then purposively selected for maximum impact. The heading ‘Sample size determination’ is important because there we explained that the study was a total population study of all persons affected by leprosy or BU in the selected LGAs.

**Reviewer’s Comment**

10.rather than saing study instrument, it is better to say data collection tools and try to merge it with data collection procedures.

**Author response**

**Correction done.**

**Reviewer’s Comment**

11. the chrosectional nature of the study and purposively selection of the sample is also the limitation of the study. please include this

**Author response**

**Included. Thanks**
